# A Proof‐of‐Concept Study of Language‐Stratified Assessment for Minimal Hepatic Encephalopathy: Integrating the Animal Naming Test and Serum IL‐6

**DOI:** 10.1002/kjm2.70271

**Published:** 2026-08-20

**Authors:** Hsin‐Che Lin, Cheng Jen Chen, Tsung‐Han Wu, Po‐Wen Gu, Chun‐Yi Tsai, Wey‐Ran Lin, Chao‐Wei Hsu, Chun‐Yen Lin, Rong‐Nan Chien, Chien‐Hao Huang, Piero Amodio

**Affiliations:** ^1^ Division of Hepatology, Department of Gastroenterology and Hepatology, Linkou Medical Center Chang Gung Memorial Hospital Taoyuan Taiwan; ^2^ College of Medicine Chang Gung University Taoyuan Taiwan; ^3^ Department of General Surgery Chang‐Gung Memorial Hospital, Linkou Medical Center Taoyuan Taiwan; ^4^ Department of Laboratory Medicine Taipei Chang Gung Memorial Hospital Taipei Taiwan; ^5^ Liver Research Center, Chang Gung Memorial Hospital Linkou Taiwan; ^6^ Department of Medicine University of Padova Padova Italy; ^7^ Private Clinic “Parco dei Tigli” Teolo Italy

**Keywords:** liver cirrhosis, minimal hepatic encephalopathy, primary spoken language, semantic verbal fluency, serum interleukin‐6

## Abstract

Minimal hepatic encephalopathy (MHE) involves subtle cognitive dysfunction and systemic inflammation and is associated with an increased risk of overt hepatic encephalopathy. The Animal Naming Test (ANT_1_) is a rapid semantic fluency tool for MHE assessment, but its performance across different primary spoken languages remains unclear. We conducted a prospective proof‐of‐concept study to evaluate the diagnostic performance of ANT_1_ and serum interleukin‐6 (IL‐6) in Mandarin‐ and Taiwanese Hokkien‐speaking cirrhotic patients. A total of 65 cirrhotic patients and 34 healthy controls were enrolled. Patients completed ANT_1_, simplified ANT_1_ (S‐ANT_1_), and standard psychometric assessments. MHE was defined by abnormal PHES and/or visually assessed EEG slowing. Diagnostic discrimination was evaluated using AUROC analyses stratified by primary spoken language. A post hoc exploratory Taiwanese‐calibrated S‐ANT_1_ was also assessed in Taiwanese Hokkien‐speaking patients. Sixteen cirrhotic patients (24.6%) were diagnosed with MHE. Patients with MHE had lower ANT1 scores and higher serum IL‐6 levels than those without MHE. In Mandarin‐speaking patients (*n* = 44), ANT_1_ demonstrated an AUROC of 0.760, while serum IL‐6 showed an AUROC of 0.841. The composite model combining ANT_1_ and serum IL‐6 showed a numerically higher AUROC of 0.895, but this improvement was not statistically significant in pairwise DeLong comparisons. In Taiwanese Hokkien‐speaking patients (*n* = 21), standard ANT_1_ showed a lower AUROC of 0.679. The exploratory Taiwanese‐calibrated S‐ANT_1_ showed a numerically higher AUROC of 0.776; however, this post hoc finding was based on a small subgroup with 7 MHE events and requires external validation. This study suggests that primary spoken language may be associated with ANT_1_ performance in MHE assessments. Integrating ANT_1_ with serum IL‐6 showed numerically improved discrimination in Mandarin speakers, whereas exploratory demographic calibration of S‐ANT_1_ showed a numerically higher AUROC in Taiwanese Hokkien speakers. These findings are exploratory and hypothesis‐generating, necessitating validation in larger independent cohorts before clinical implementation.

**Abbreviations**
ANT_1_
: animal naming test
AUROC: area under the receiver operating characteristic curve
EEG: electroencephalography
exploratory Taiwanese‐calibrated S‐ANT_1_
: exploratory Taiwanese‐calibrated simplified animal naming test
HCC: hepatocellular carcinoma
HE: hepatic encephalopathy
IL‐6: interleukin‐6
MHE: minimal hepatic encephalopathy
MMSE: mini‐mental state examination
PHES: psychometric hepatic encephalopathy score
S‐ANT_1_
: simplified animal naming test
WHC: West Haven criteria

## 1 Introduction

Hepatic encephalopathy (HE) is a serious neurological‐psychiatric complication caused by liver failure or portosystemic shunting with a broad spectrum of clinical manifestations from confusion, neurophysiological alterations, to subclinical cognitive impairment and coma [1].

Minimal hepatic encephalopathy (MHE) presents with slight or equivocal clinical findings, often lacking clear disorientation or asterixis, and is frequently underdiagnosed [1, 2]. Detecting MHE is challenging due to the minimal nature of cognitive impairment; yet, early diagnosis is crucial as MHE is associated with a higher risk of progression to overt HE (OHE) [3], increased socioeconomic burden [4], impaired driving disabilities [5], poor health‐related quality‐of‐life [6], and adverse disease outcome [7]. Therefore, identifying MHE prior to the onset of overt HE allows for early intervention, potentially reducing the burden on patients, caregivers, and the healthcare system.

Various methods have been proposed to detect MHE. Currently, the Psychometric Hepatic Encephalopathy Score (PHES) is the most validated diagnostic tool [1]; however, it is time‐consuming and relies on paper‐and‐pencil tests that are difficult to perform at the bedside, making it impractical for routine clinical practice. In terms of biomarkers, serum interleukin‐6 (IL‐6), a marker of systemic inflammation, has shown promise in diagnosing and predicting HE [8]. Elevated IL‐6 levels distinguish patients with MHE from those without, with a proposed diagnostic cutoff of > 11 pg/mL [9]. Furthermore, stepwise algorithms incorporating IL‐6 have been suggested to streamline MHE diagnosis [10].

The Animal Naming Test (ANT_1_) is a rapid, semantic verbal fluency test proposed as a first‐line screening tool for covert HE (CHE) and for monitoring patients' cognitive performance [11]. It is a routinely used method in Italy [12] and Germany [13] and has recently been recommended by the European Association for the Study of the Liver (EASL) for identifying patients with MHE or CHE [14]. While a previous Taiwanese study demonstrated the feasibility of ANT_1_ in detecting and quantifying CHE in clinical practice [15], the influence of primary spoken language and linguistic‐cultural context on its diagnostic performance remains unclear. Previous studies have suggested that semantic verbal fluency may vary across languages and may be influenced by factors such as lexical density, frequency‐of‐use distribution, linguistic structure, lexical access, and language‐use context [16]. Because semantic verbal fluency may be influenced by these linguistic factors, we explored whether primary spoken language was associated with ANT_1_ performance in a bilingual Taiwanese cohort. This language‐stratified analysis was exploratory and hypothesis‐generating. This issue is particularly relevant in Taiwan, where both Mandarin and Taiwanese Hokkien are commonly spoken by individuals at risk of cirrhosis. Moreover, the potential utility of integrating ANT_1_ with serum IL‐6 levels for MHE assessment has not been fully explored.

To address these gaps, we conducted a prospective proof‐of‐concept study with three primary aims: (1) to evaluate the diagnostic performance of ANT_1_ in identifying MHE in cirrhotic patients, (2) to explore whether primary spoken language, Mandarin versus Taiwanese Hokkien, is associated with ANT_1_ diagnostic performance, and (3) to evaluate the potential added diagnostic value of serum IL‐6 in the assessment of MHE.

## 2 Materials and Methods

### 2.1 Study Design and Patient Enrolment

A single‐center prospective cohort study was conducted at a tertiary referral hospital in Taiwan to evaluate the diagnostic performance of the ANT_1_ stratified by primary spoken language (Mandarin vs. Taiwanese Hokkien) for detecting MHE among consecutive outpatients with cirrhosis. Cirrhosis was diagnosed based on clinical, biochemical, and ultrasonographic/CT/MRI or pathology criteria. The standardized ANT1 (S‐ANT_1_), an age‐ and education‐adjusted version validated in Italy [11], was also analyzed. All participants underwent ANT_1_, the Psychometric Hepatic Encephalopathy Score (PHES), and the Mini‐Mental State Examination (MMSE) on the same day; the patients underwent an eyes‐closed digitalized electroencephalogram (EEG), which was visually assessed by an expert electroencephalographer for signs of slowing compatible with HE [17, 18]. Age‐matched healthy controls were recruited from relatives of cirrhotic patients.

**FIGURE 1:**
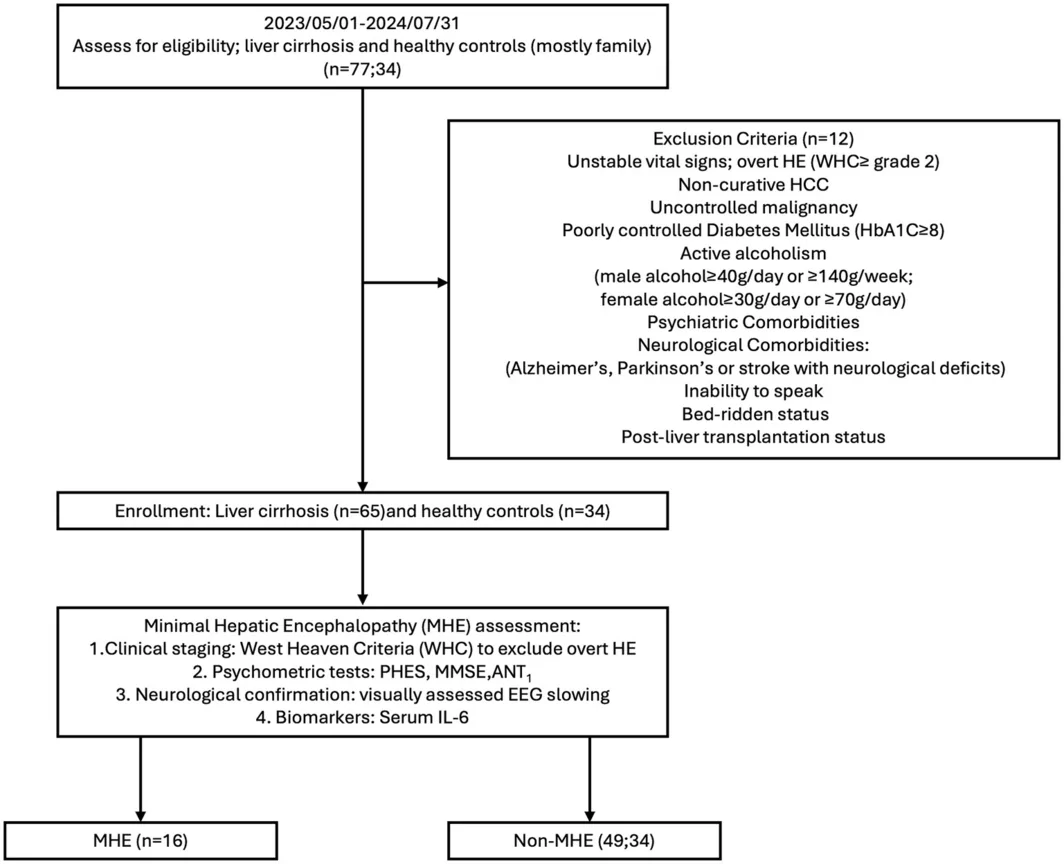
Flowchart of patient recruitment and study design. The study was conducted between May 1, 2023, and July 31, 2024. A total of 77 patients with liver cirrhosis and 34 healthy controls were assessed for eligibility. All participants underwent a comprehensive MHE Assessment Protocol, which included: (1) Clinical staging using West Haven Criteria (WHC) to exclude overt HE; (2) Psychometric testing (PHES, ANT1, MMSE); (3) Neurophysiological confirmation via Quantitative EEG; and (4) Biomarker analysis (Serum IL‐6). After excluding 12 patients based on strict exclusion criteria (e.g., overt HE grade ≧ 2, neurological comorbidities, or uncontrolled metabolic diseases), a total of 65 cirrhotic patients were enrolled and stratified into MHE (*n* = 16) and Non‐MHE (*n* = 49) groups. Thirty‐four age‐matched healthy controls were included for comparison.

Exclusion criteria included: age < 20 years; neurologic or psychiatric comorbidities (e.g., Alzheimer's disease, Parkinson's disease, cerebrovascular disease); alcoholic dementia (Wernicke encephalopathy, Korsakoff syndrome); active alcohol misuse or substance abuse within 6 months; liver transplantation within 1 year; advanced hepatocellular carcinoma (BCLC stage ≥ B); active extrahepatic malignancy; unstable vital signs; recent hospitalization for acute decompensation; cardiac failure; or respiratory insufficiency (Figure 1, flowchart).

### 2.2 Relationship With the Previous ANT_1_ Cohort

The present cohort partially overlapped with our previous 2023 ANT_1_ study [15]. Specifically, of the 65 cirrhotic patients in the present study, 13 were also included in the previous cohort, whereas 52 were newly recruited. Some patients from the previous cohort were not included in the present analysis because of death, liver transplantation, loss to follow‐up, enrollment in other ongoing protocols that precluded participation in the present study, or failure to meet the eligibility criteria for the present protocol. The previous study primarily focused on the feasibility and cutoff of ANT_1_ for detecting CHE in a Chinese‐speaking Taiwanese population, whereas the present study specifically evaluated language‐stratified ANT_1_ performance and serum IL‐6 in the assessment of MHE.

### 2.3 Definition of MHE

MHE was defined using a composite reference standard consisting of abnormal PHES and/or visually assessed EEG slowing compatible with hepatic encephalopathy, in patients without clinically overt HE according to the West Haven criteria. An abnormal PHES was defined as a total score of ≤ −4. PHES was calculated using the Hannover Medical School algorithm, and this threshold was selected because it is consistent with the Hannover PHES approach and with our previous Chinese‐speaking ANT1 validation study. EEG slowing/abnormality was defined as a dominant posterior background rhythm < 8 Hz and/or clearly increased diffuse theta/delta activity beyond age‐expected background activity, as interpreted by an experienced neurophysiologist blinded to clinical and psychometric data. No quantitative spectral threshold or percentage‐of‐recording criterion was applied [1, 18, 19].

### 2.4 Psychometric Test Scoring

ANT_1_ is a semantic verbal fluency test in which participants are instructed to name as many animals as possible within 1 min. The test was administered in either Mandarin or Taiwanese Hokkien according to the participant's primary spoken language. Primary spoken language was defined as the participant's self‐reported dominant language for daily communication and the language selected for ANT_1_ administration. The assigned language group was confirmed with each participant by a trained study coordinator before testing. This classification was intended as a pragmatic clinical grouping based on dominant daily language use and did not imply monolingual status.

The S‐ANT_1_ score, as proposed by Campagna et al. and our previous preliminary study, was calculated by adding 3 points for individuals with fewer than 8 years of education and an additional 3 points, totaling 6 points, for those aged over 80 years [11, 15]. Because the Taiwanese Hokkien‐speaking subgroup had distinct age and education distributions, we also performed an exploratory post hoc demographic calibration of S‐ANT_1_ by adding three points for age > 70 years and/or education < 6 years. This score was termed the exploratory Taiwanese‐calibrated S‐ANT_1_. Given that this calibration was derived from the same study sample, bootstrap internal validation was performed within the Taiwanese Hokkien‐speaking subgroup to estimate the optimism‐corrected area under the receiver operating characteristic curve (AUROC). The exploratory Taiwanese‐calibrated S‐ANT_1_ was considered hypothesis‐generating and was not regarded as an externally validated score.

MMSE was performed following standard protocols [20]. PHES was calculated using the Hannover Medical School algorithm [21]. PHES ≤ −4 was selected because it is consistent with the Hannover PHES approach and with our previous Chinese‐speaking ANT_1_ validation study, while acknowledging that other thresholds such as < −4 or ≤ −5, have been used in different normative datasets. PHES was performed once at baseline during the same study visit as ANT1 and MMSE, and the baseline PHES result was used for MHE classification. Repeated PHES testing was not performed on the same day to avoid potential learning effects. Serial PHES assessment was performed during the 3‐month follow‐up period for longitudinal evaluation, but these follow‐up PHES results were not used to define baseline MHE status. All PHES tests were administered by the same trained tester using a standardized protocol.

### 2.5 Serum IL‐6 Quantification

Blood samples for serum IL‐6 measurement were collected during the same study visit as the PHES, ANT_1_, and MMSE assessments. Serum was separated and stored at −80°C until analysis.

Serum interleukin‐6 (IL‐6) concentrations were measured using an electrochemiluminescence immunoassay (ECLIA) on the Roche cobas platform (Roche Diagnostics, Mannheim, Germany) according to the manufacturer's protocol. Blood samples were collected in serum‐separation tubes, centrifuged at 1500 × g for 10 min, and the serum was stored at −80°C until analysis. All measurements were performed in a CAP‐certified clinical central laboratory, with identical quality‐control procedures applied to all runs.

### 2.6 Statistical Analysis

Continuous variables were expressed as mean ± SD for normally distributed data or median (IQR) for skewed distributions. Normality was assessed using the Shapiro–Wilk test. Between‐group differences were analyzed using Student's *t*‐test or the Wilcoxon rank‐sum test, as appropriate. Categorical variables were presented as counts (percentages) and compared using the Chi‐square or Fisher's exact test. To explore whether ANT_1_ diagnostic performance differed by primary spoken language, ROC analyses were performed separately in Mandarin‐ and Taiwanese Hokkien‐speaking patients. To evaluate whether ANT_1_ was associated with MHE independent of demographic factors and primary spoken language, we performed multivariable logistic regression with MHE status as the dependent variable and age, education years, primary spoken language, and ANT_1_ score as independent variables. This model was also used to assess whether primary spoken language was independently associated with MHE after adjustment for age, education years, and ANT_1_ score. Language‐stratified multivariable logistic regression models were further performed in Mandarin‐ and Taiwanese Hokkien‐speaking patients, with age, education years, and ANT_1_ score included as independent variables. ROC curves were constructed, and AUROCs were calculated to evaluate diagnostic performance. Comparisons between AUCs were performed using DeLong's test. To evaluate the combined diagnostic performance of ANT_1_ and serum IL‐6, we constructed a diagnostic composite model using binary logistic regression with MHE status as the dependent variable. ANT_1_ scores and serum IL‐6 levels were entered as independent covariates to generate predicted probabilities for the composite model. For exploratory subgroup comparisons, multiplicity was assessed using Benjamini‐Hochberg FDR correction; pairwise AUROC comparisons were additionally interpreted after Holm‐Bonferroni correction. All statistical analyses were conducted using SAS Studio and GraphPad Prism. Two‐tailed *p*‐values < 0.05 were considered statistically significant.

### 2.7 Ethical Clearance

The study was approved by the Institutional Review Board of Chang Gung Memorial Hospital, Linkou Branch (202002431B0A3 and 202302207A3W001). Written informed consent was obtained from all participants. The study adhered to the principles of the Declaration of Helsinki.

## 3 Results

### 3.1 Baseline Characteristics Comparisons Between Patients With and Without Minimal Hepatic Encephalopathy

A total of 65 patients with liver cirrhosis and 34 healthy controls were enrolled (Table 1). Among cirrhotic patients, 16 (24.6%) were diagnosed with MHE (Figure 1). Patients with MHE were significantly older than those without (65.6 vs. 59.6 years, *p* = 0.032), while sex distribution (68.7% vs. 65.3% male, *p* = 0.800) and cirrhosis etiology (*p* = 0.954) were comparable. Years of education were also similar (median 9 vs. 12 years, *p* = 0.138).

**TABLE 1:** Basic characteristics of participants in the cohort.

	Total patients (*N* = 99)	Subgroup			*p* *
		Healthy control (*N* = 34)	Hepatic encephalopathy		
			Non‐MHE (*N* = 49)	MHE (*N* = 16)	
Age	60.8 ± 9.5	61.0 ± 10.8	59.6 ± 9.7	65.6 ± 7.8	0.032^a^
BMI	25.5 ± 3.5	25.5 ± 2.2	25.4 ± 3.5	26.0 ± 3.6	0.638^a^
Gender					0.800^a^
Male	58 (58.6)	15 (44.1)	32 (65.3)	11 (68.7)	
Female	41 (41.4)	19 (55.9)	17 (34.7)	5 (31.3)	
Etiology					0.954
HBV	37 (56.9)		28 (57.1)	9 (56.3)	
HCV	9 (13.9)		7 (14.3)	2 (12.5)	
Alcohol	17 (26.2)		12 (24.5)	5 (31.2)	
NASH	1 (1.5)		1 (2.0)		
Others	1 (1.5)		1 (2.0)		
Education years					0.138
	12 (9–14)	12 (9–16)	12 (9–12)	9 (6–12)	
Language					0.260
Mandarin	64 (64.6)	20 (58.8)	35 (71.4)	9 (56.2)	
Taiwanese	35 (35.4)	14 (41.2)	14 (28.6)	7 (43.8)	
Psychometric tests					
PHES	0 (−2 to 1)	(−0.5) (−2 to 1.8)	0 (−1 to 2)	(−2) (−4.3 to 0)	0.001
MMSE	28 (27–29)	29 (27–30)	28 (26–29)	26.5 (23.5–29)	0.052
ANT_1_	18 (14.5–22)	19 (16.3–22.8)	19 (14–22)	15 (12–16.5)	0.014
S‐ANT_1_	19 (16–22)	19.5 (17.3–23)	19 (15–22)	15.5 (13.5–16.5)	0.023
Exploratory Taiwanese‐ calibrated S‐ANT_1_	19 (16–22.5)	21 (18.3–23)	20 (16–23)	15.5 (12–18)	0.0035
Serum IL‐6			3.1 (2–4.4)	7.2 (4.4–10)	< 0.001

* *p*‐value compares **Non‐MHE versus MHE** groups.

^a^ There were no significant differences in age or BMI, or gender distribution between healthy controls and the total cirrhotic cohort (*p* > 0.05).

**FIGURE 2:**
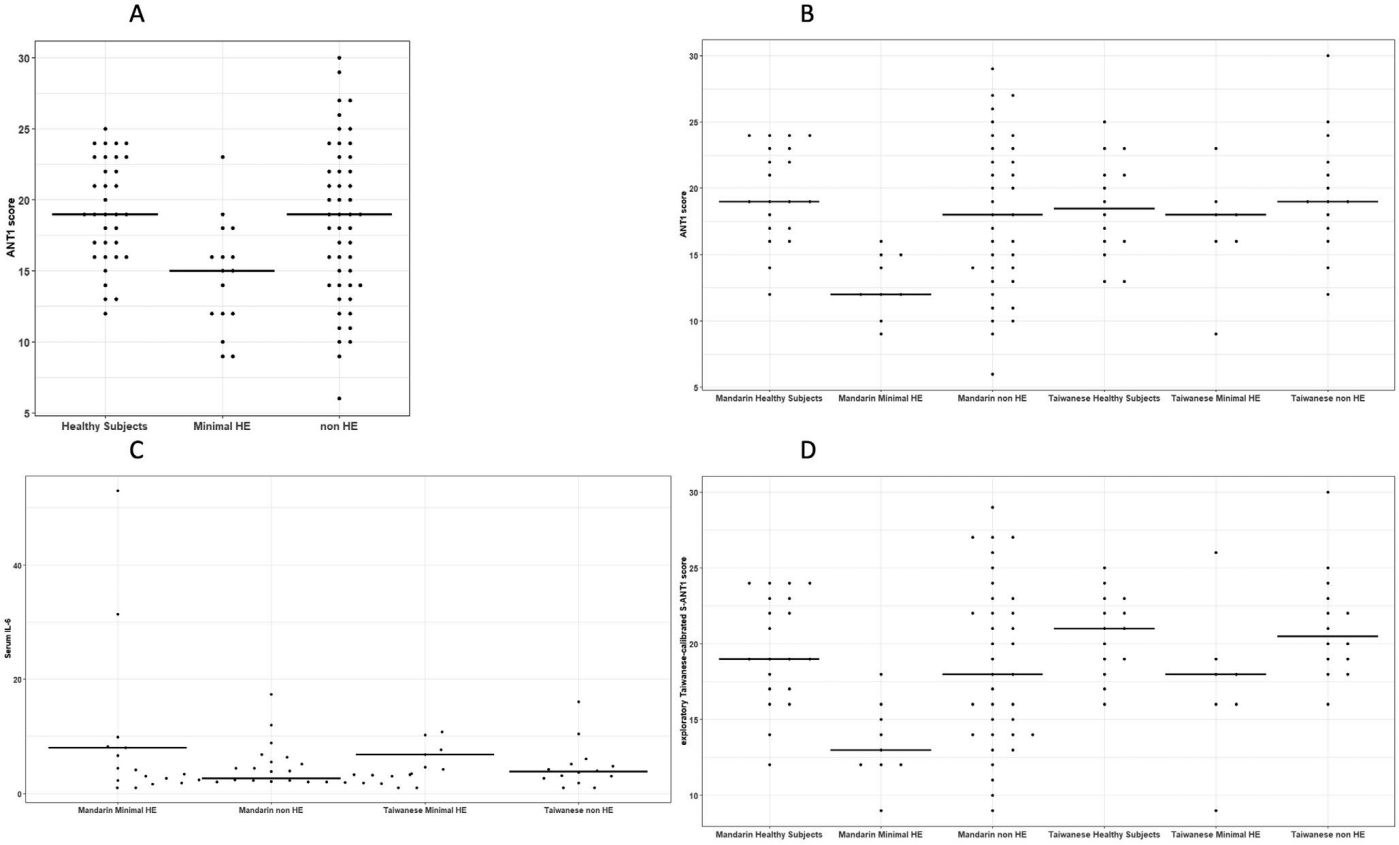
Distribution of psychometric scores and serum IL‐6 levels according to MHE status and primary spoken language. (A) In the overall cohort, ANT_1_ scores were significantly lower in patients with minimal hepatic encephalopathy (MHE) than in non‐MHE patients (*p* = 0.014). Healthy controls are shown for reference. (B) In the language‐stratified analysis, ANT_1_ scores were significantly lower in MHE patients than in non‐MHE patients among Mandarin speakers (*p* = 0.017), whereas no significant between‐group difference was observed among Taiwanese Hokkien‐speaking patients (*p* = 0.189). (C) Serum IL‐6 levels were significantly elevated in Mandarin‐speaking MHE patients compared to non‐MHE patients (*p* = 0.002). In Taiwanese Hokkien‐speaking patients, IL‐6 levels were numerically higher in MHE patients, but the difference did not reach statistical significance (*p* = 0.086). (D) Distribution of scores using the exploratory Taiwanese‐calibrated S‐ ANT_1_, defined by adding three points for age > 70 years and/or education < 6 years. The exploratory Taiwanese‐calibrated S‐ ANT_1_ showed a nominal between‐group difference between MHE and non‐MHE patients in the Taiwanese Hokkien‐speaking subgroup (*p* = 0.043). This post hoc finding should be interpreted as exploratory. Horizontal lines represent the median values.

Neurocognitive assessments showed significantly lower scores in the MHE group for PHES (−2 vs. 0, *p* = 0.001), ANT_1_ (15.0 vs. 19.0, *p* = 0.014, Figure 2A), and S‐ANT_1_ (15.5 vs. 19, *p* = 0.023). MMSE scores were slightly lower in the MHE group but did not reach statistical significance (26.5 vs. 28, *p* = 0.052). Serum IL‐6 levels were significantly higher in patients with MHE compared to those without (7.2 vs. 3.1 pg/mL, *p* < 0.001).

### 3.2 Subgroup Analysis of Psychometric Scores and Serum IL‐6 by Primary Spoken Language

To explore whether psychometric and biomarker performance differed by primary spoken language, we compared Mandarin‐ and Taiwanese Hokkien‐speaking cirrhotic patients with and without MHE. In the Mandarin‐speaking subgroup, patients with MHE had significantly lower ANT_1_ scores than non‐MHE patients (median 12 vs. 18, *p* = 0.017, Table 2A, Figure 2B). Similar significant differences were consistently observed for S‐ANT_1_ (15 vs. 18, *p* = 0.020), PHES (−2 vs. 1, *p* < 0.001), and MMSE (26 vs. 29, *p* = 0.029) (Table 2A). Serum IL‐6 levels were also significantly higher in Mandarin‐speaking patients with MHE than in those without MHE (8.0 vs. 2.7 pg/mL, *p* = 0.002, Figure 2C). After Benjamini‐Hochberg FDR correction across the 12 psychometric and biomarker comparisons, ANT_1_, S‐ANT_1_, exploratory Taiwanese‐calibrated S‐ANT_1_, PHES, and serum IL‐6 remained significantly different in Mandarin‐speaking patients, whereas MMSE did not.

**TABLE 2:** Psychometric tests' performance in predicting minimal hepatic encephalopathy in different populations.

(A) Mandarin‐speaking populations					
	Total (*N* = 44)	Non‐MHE (*N* = 35)	MHE (*N* = 9)	*p*	FDR adjusted *p*‐value
Age				0.087	
	59.4 ± 9.9	58.1 ± 9.8	64.4 ± 9.3		
BMI				0.874	
	25.6 ± 3.4	25.5 ± 3.5	25.7 ± 3.3		
Gender				0.695	
Male	29 (65.9)	22 (62.9)	7 (77.8)		
Female	15 (34.1)	13 (37.1)	2 (22.2)		
Education years				0.268	
	12 (9–12)	12 (9–12)	9 (9–12)		
Psychometric tests					
ANT_1_	15.5 (12–21.3)	18 (13.5–22.5)	12 (12–15)	0.017	0.049
S‐ANT_1_	16 (14–21.3)	18 (14–22.5)	15 (12–15)	0.020	0.049
Exploratory Taiwanese‐ calibrated S‐ANT_1_	16 (13.8–22)	18 (14–22.5)	13 (12–15)	0.007	0.029
PHES	0 (−1 to 1)	0 (−1 to 2)	−2 (−4 to (−1))	< 0.001	0.008
MMSE	29 (26–29)	29 (27–29.5)	26 (23–29)	0.029	0.058
Serum IL‐6	3.2 (2.0–5.7)	2.7 (1.9–4.2)	8.0 (4.4–9.9)	0.002	0.011

(B) Taiwanese Hokkin‐speaking populations					
	Total (*N* = 21)	Non‐MHE (*N* = 14)	MHE (*N* = 7)	*p*	FDR adjusted *p*‐value
Age	64.5 ± 8.0	63.3 ± 8.8	67.0 ± 5.8	0.328	
BMI	25.4 ± 3.9	24.9 ± 3.7	26.3 ± 4.3	0.481	
Gender				0.638	
Male	14 (66.7)	10 (71.4)	4 (57.1)		
Female	7 (33.3)	4 (28.6)	3 (42.9)		
Education years				0.544	
	9 (6–12)	9 (8.3–12)	9 (6–10.5)		
Psychometric tests					
ANT_1_	19 (16–22)	19 (17.3–21.8)	18 (16–18.5)	0.189	0.252
S‐ANT_1_	19 (17–22)	19.5 (18.3–21.8)	18 (16–21.5)	0.389	0.467
Exploratory Taiwanese‐ calibrated S‐ANT_1_	19 (18–22)	20.5 (19–22.8)	18 (16–18.5)	0.043	0.073
PHES	−1 (−3 to 0.3)	−1 (−2 to 1)	−2 (−5 to 0)	0.448	0.489
MMSE	27 (25–29)	27 (25.3–27.8)	27 (24.5–29)	0.970	0.970
Serum IL‐6	4.2 (3.1–6.8)	3.8 (2.7–5)	6.8 (4.4–8.9)	0.086	0.129

*Note:* Benjamini–Hochberg false‐discovery‐rate correction was applied across the 12 psychometric and biomarker comparisons derived from six variables across the two language subgroups. Baseline demographic variables were not included in this FDR correction.

In contrast, this discriminatory ability was attenuated in the Taiwanese Hokkien‐speaking subgroup (Table 2B). Standard ANT_1_ (18 vs. 19, *p* = 0.189) and S‐ANT_1_ (18 vs. 19.5, *p* = 0.389), PHES (−2 vs. −1, *p* = 0.448), and MMSE (27 vs. 27, *p* = 0.970) were not significantly different between MHE and non‐MHE patients. Serum IL‐6 levels were numerically higher in MHE patients than in non‐MHE patients (6.8 vs. 3.8 pg/mL), but this difference did not reach statistical significance (*p* = 0.086, Figure 2C). The exploratory Taiwanese‐calibrated S‐ANT_1_ showed a nominal between‐group difference before FDR correction (18 vs. 20.5, *p* = 0.043), but this did not remain significant after correction (*p* = 0.073). However, no biomarkers or psychometric scores were all non‐significant in the Taiwanese Hokkien‐speaking subgroup. Overall, no psychometric or biomarker comparison remained statistically significant after FDR correction in the Taiwanese Hokkien‐speaking subgroup.

### 3.3 Multivariable Analysis of Factors Associated With MHE

To evaluate whether the association between ANT_1_ and MHE was independent of demographic factors, we performed multivariable logistic regression including age, education years, primary spoken language, and ANT_1_ score in the total cirrhotic cohort. ANT_1_ score remained independently associated with MHE after adjustment for age, education years, and primary spoken language (OR 0.796, 95% CI: 0.654–0.969, *p* = 0.023; Table S1A). Age, education years, and primary spoken language were not independently associated with MHE after adjustment. In language‐stratified analyses, ANT_1_ remained significantly associated with MHE in Mandarin‐speaking patients after adjustment for age and education years (OR 0.744, 95% CI: 0.568–0.974, *p* = 0.032; Table S1B). In contrast, ANT_1_ was not significantly associated with MHE in Taiwanese Hokkien‐speaking patients after adjustment for age and education years (OR 0.868, 95% CI: 0.648–1.164, *p* = 0.345; Table S1C). Given the small number of Taiwanese Hokkien‐speaking patients and MHE events, this subgroup result should be interpreted cautiously.

We further performed an exploratory age‐stratified analysis by dividing patients into younger and older age groups. ANT_1_ performance appeared attenuated in older patients, with an AUROC of 0.820 in patients aged < 65 years and 0.528 in patients aged ≥ 65 years (Table S2). Because of the limited number of MHE events within each age stratum, these subgroup analyses should be interpreted as exploratory. Table S3 compares the baseline characteristics of Mandarin and Taiwanese Hokkien speakers, including MHE prevalence, sex, EEG slowing, cirrhosis etiology, age, education years, MELD score, PHES, ANT_1_, S‐ANT_1_, MMSE, and serum IL‐6 levels. Taiwanese Hokkien speakers tended to be older and had fewer years of education, although these differences did not reach statistical significance.

### 3.4 Diagnostic Performance of ANT_1_ , S‐ANT_1_ , Exploratory Taiwanese‐Calibrated S‐ANT_1_ , Serum IL‐6, and the Composite Model for MHE

**FIGURE 3:**
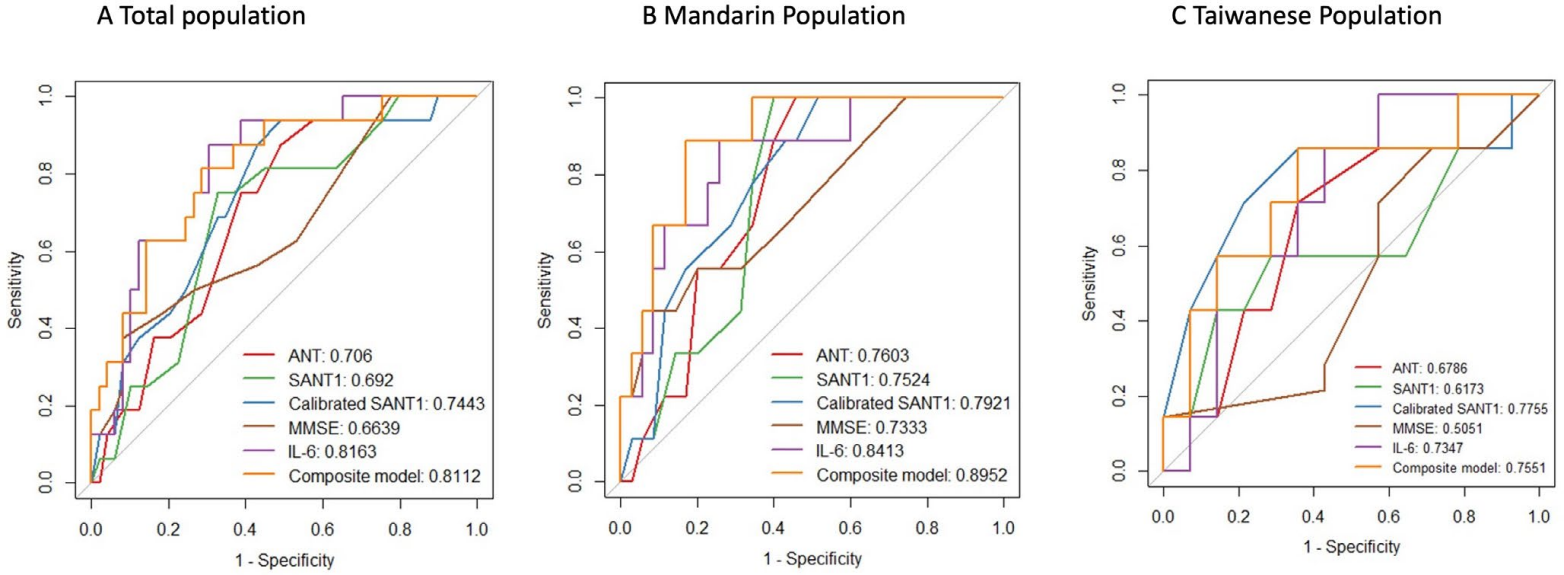
Receiver operating characteristic (ROC) curves illustrating the diagnostic performance of psychometric tests, serum IL‐6, and the composite model for detecting minimal hepatic encephalopathy (MHE). (A) In the overall cohort, serum IL‐6 showed the highest AUROC among the single markers (AUROC 0.816), followed by ANT_1_ (AUROC 0.706), S‐ANT1 (AUROC 0.692), and MMSE (AUROC 0.664). (B) In Mandarin‐speaking patients, serum IL‐6 showed an AUROC of 0.841, and ANT_1_ showed an AUROC of 0.760. The composite model combining ANT_1_ and serum IL‐6 showed a numerically higher AUROC of 0.895; however, pairwise DeLong comparisons did not show statistically significant differences between the composite model and either ANT_1_ or serum IL‐6 alone. (C) In Taiwanese Hokkien‐speaking patients, the AUROCs of standard ANT_1_ and S‐A ANT_1_ were 0.679 and 0.617, respectively. The exploratory Taiwanese‐calibrated S‐ ANT_1_, defined by adding three points for age > 70 years and/or education < 6 years, showed a numerically higher AUROC of 0.776. Adding serum IL‐6 to the exploratory Taiwanese‐calibrated S‐ ANT_1_ did not numerically improve the AUROC in this small subgroup. These subgroup findings should be interpreted as exploratory. ANT_1_, animal naming test; AUROC, area under the receiver operating characteristic curve; Calibrated S‐ANT_1_, Taiwanese‐specific calibrated S‐ANT_1_; IL‐6, interleukin‐6; MMSE, mini‐mental state examination; S‐ANT_1_, standardized ANT_1_.

The diagnostic performance of ANT_1_, S‐ANT_1_, exploratory Taiwanese‐calibrated S‐ANT_1_, MMSE, serum IL‐6, and the composite model for identifying MHE is shown in Table 3 and Figure 3. In the overall cohort, serum IL‐6 showed the highest AUROC among the single markers, with an AUROC of 0.816. The cohort‐specific Youden cutoff for IL‐6 was 4.1 pg/mL, yielding a sensitivity of 0.875 and specificity of 0.694. To improve clinical interpretability and address previously reported IL‐6 thresholds, we further evaluated fixed cutoffs of 7, 8, and 11 pg/mL. Sensitivity decreased from 0.875 at the cohort‐specific cutoff to 0.500 at 7 pg/mL, 0.438 at 8 pg/mL, and 0.125 at 11 pg/mL, whereas specificity increased from 0.694 to 0.898, 0.898, and 0.939, respectively. Sensitivity, specificity, positive predictive value (PPV), and negative predictive value (NPV) for each threshold are shown in Table S4.

**TABLE 3:** The AUROC of ANT_1_, S‐ANT_1_, Taiwan S‐ANT_1_, MMSE and Serum IL‐6 in detecting minimal Hepatic encephalopathy.

(A) Total populations							
AUROC (95% CI)		Pairwise comparison for AUROC (*p*)					
		ANT_1_	S‐ANT_1_	Taiwan S‐ANT_1_	MMSE	Serum IL‐6	New model
ANT_1_	0.706 (0.573–0.839)						
S‐ANT_1_	0.692 (0.550–0.834)	0.6526					
Exploratory Taiwanese‐ calibrated S‐ANT_1_	0.744 (0.610–0.879)	0.1285	0.1237				
MMSE	0.664 (0.508–0.820)	0.6225	0.7821	0.3706			
Serum IL‐6	0.816 (0.706–0.927)	0.1981	0.1628	0.4174	0.1244		
Composite model	0.811 (0.693–0.930)	0.2494	0.2090	0.4656	0.1427	0.9509	

(B) Mandarin‐speaking populations							
AUROC (95% CI)		Pairwise comparison for AUROC (*p*)					
		ANT_1_	S‐ANT_1_	Taiwan S‐ANT_1_	MMSE	Serum IL‐6	Newmodel
ANT_1_	0.760 (0.619–0.902)						
S‐ANT_1_	0.752 (0.609–0.896)	0.8632					
Exploratory Taiwanese‐ calibrated S‐ANT_1_	0.792 (0.652–0.932)	0.3270	0.4145				
MMSE	0.733 (0.545–0.922)	0.7890	0.8715	0.5908			
Serum IL‐6	0.841 (0.699–0.984)	0.4395	0.4078	0.6505	0.4168		
Composite model	0.895 (0.797–0.993)	0.1286	0.1119	0.2410	0.1407	0.5424	

(C) Taiwanese Hokkien‐speaking populations							
AUROC (95% CI)		Pairwise comparison for AUROC (*p*)					
		ANT_1_	S‐ANT_1_	Taiwanese S‐ANT_1_	MMSE	Serum IL‐6	Composite model
ANT_1_	0.679 (0.432–0.926)						
S‐ANT_1_	0.617 (0.330–0.904)	0.4478					
Exploratory Taiwanese‐ calibrated S‐ANT_1_	0.776 (0.516–1.000)	0.2299	0.1364				
MMSE	0.505 (0.212–0.798)	0.3805	0.5947	0.1831			
Serum IL‐6	0.735 (0.513–0.957)	0.7334	0.4825	0.8229	0.2289		
Composite model	0.755 (0.517–0.993)	0.6643	0.4734	0.9101	0.2023	0.9029	

*Note:* The composite model included ANT_1_ and serum IL‐6. Pairwise AUROC comparisons were performed using DeLong's test. All pairwise comparisons were non‐significant before multiple‐testing correction and remained non‐significant after Holm–Bonferroni correction. Therefore, raw DeLong *p* values are presented.

Abbreviations: AUROC, area under the receiver operating characteristic curve; CI, confidence interval.

Among psychometric tests in the overall cohort, ANT_1_ showed the highest AUROC (0.706), followed by S‐ANT_1_ (0.692) and MMSE (0.664). In Mandarin‐speaking patients (Table 3B and Figure 3B), serum IL‐6 showed a high AUROC of 0.841, and ANT_1_ showed an AUROC of 0.760. The composite model combining ANT_1_ and serum IL‐6 showed a numerically higher AUROC than either ANT_1_ or serum IL‐6 alone. However, pairwise DeLong comparisons did not show statistically significant differences between the composite model and either ANT_1_ or serum IL‐6 alone.

In Taiwanese Hokkien‐speaking patients (Table 3C and Figure 3C), the discriminatory performance of standard psychometric measures was lower: AUROCs were 0.679 for ANT_1_, 0.617 for S‐ANT_1_, and 0.505 for MMSE. Serum IL‐6 showed an AUROC of 0.735 in this subgroup.

To address potential incorporation bias related to the use of PHES in the composite MHE reference standard, we further performed a sensitivity analysis using EEG‐defined MHE as the reference condition. In this analysis, ANT_1_, S‐ANT_1_, exploratory Taiwanese‐calibrated S‐ANT_1_, MMSE, serum IL‐6, and the composite model retained moderate discriminatory performance, with AUROCs ranging from 0.699 to 0.766 (Table S5). Because only 13 patients were EEG‐positive, this sensitivity analysis was performed in the total cohort only, was not further stratified by primary spoken language, and should be interpreted as exploratory.

### 3.5 Exploratory Calibration of ANT_1_ in Taiwanese Hokkien‐Speaking Participants

To explore whether demographic adjustment might improve discrimination, we applied an exploratory calibration based on the S‐ANT_1_ framework by adding three points to ANT_1_ scores for patients aged > 70 years and/or with fewer than 6 years of education (Figure 2D). This strategy, termed the exploratory Taiwanese‐calibrated S‐ANT_1_, showed a nominal between‐group difference between MHE and non‐MHE patients in the Taiwanese Hokkien‐speaking subgroup before FDR correction (*p* = 0.043, Table 2B), but this difference did not remain significant after correction (FDR‐adjusted *p* = 0.073). In ROC analysis, the exploratory Taiwanese‐calibrated S‐ANT_1_ showed a numerically higher AUROC than the original S‐ANT_1_ in Taiwanese Hokkien‐speaking patients (0.776 vs. 0.617, Figure 3C), but this difference did not reach statistical significance by DeLong's test (*p* = 0.136). Adding IL‐6 to the exploratory Taiwanese‐calibrated S‐ANT_1_ did not numerically improve AUROC in this subgroup (composite model AUROC 0.755). Given the small subgroup size and post hoc nature of the calibration, these findings should be interpreted as exploratory.

Because the exploratory Taiwanese‐calibrated S‐ANT_1_ was derived from the same Taiwanese Hokkien‐speaking subgroup, we performed bootstrap internal validation to assess optimism. The apparent AUROC of the exploratory Taiwanese‐calibrated S‐ANT_1_ was 0.776. After bootstrap correction for optimism, the AUROC was 0.773 (Table S6). This bootstrap analysis represents internal validation only and should not be interpreted as external validation.

## 4 Discussion

In this prospective proof‐of‐concept study, we evaluated the interplay among psychometric performance, primary spoken language, and systemic inflammation in the assessment of MHE. To our knowledge, few studies have explored whether primary spoken language may be associated with ANT_1_ performance in a bilingual East Asian clinical setting. Our principal findings are threefold: (1) standard ANT_1_ and S‐ANT_1_ appeared to show better diagnostic performance in Mandarin‐speaking patients than in Taiwanese Hokkien‐speaking patients; (2) integrating ANT_1_ with serum IL‐6 showed a numerically higher AUROC in Mandarin‐speaking patients (AUROC 0.895), although this improvement was not statistically significant in pairwise DeLong comparisons; and (3) in Taiwanese Hokkien‐speaking patients, the post hoc exploratory Taiwanese‐calibrated S‐ANT_1_ showed a numerical improvement in discrimination, although this finding requires external validation.

The EASL guideline recommends ANT_1_ as a practical tool for identifying patients with MHE or CHE, with routine application reported in Italy and Germany [1, 12, 13]. Our findings extend these observations to an East Asian cohort and suggest that both ANT_1_ and the Italian standardized S‐ANT_1_ retain diagnostic utility in Mandarin‐speaking patients. This aligns with prior Taiwanese data supporting ANT_1_/S‐ANT_1_ for CHE detection [15], as well as a Chinese study showing that S‐ANT_1_ can identify MHE in Mandarin‐speaking regions [22]. An exploratory finding of our study is the numerically higher AUROC observed when combining ANT_1_ with serum IL‐6 in Mandarin speakers (AUROC 0.895). Although IL‐6 has been reported as a marker associated with HE‐related neuroinflammation [8, 9], integrating ANT_1_ with serum IL‐6 did not show statistically significant improvement in pairwise DeLong comparisons compared with either ANT_1_ or serum IL‐6 alone. Therefore, the potential added value of combining psychometric and inflammatory markers should be interpreted as exploratory and hypothesis‐generating rather than as evidence of a synergistic effect or a validated diagnostic algorithm.

In contrast, the diagnostic performance of standard ANT_1_ appeared attenuated in Taiwanese Hokkien speakers. This finding suggests that applying a single psychometric cutoff across linguistically and demographically diverse populations may be problematic, although this observation should be interpreted cautiously given the small subgroup size. Taiwan provides a relevant setting to explore this issue because Mandarin and Taiwanese Hokkien are commonly used in daily communication. Prior cross‐linguistic and bilingual neuropsychological studies have suggested that language background, bilingual language use, vocabulary knowledge, and cross‐linguistic differences may influence semantic verbal fluency performance [23, 24, 25]. Cross‐national studies have also reported variable ANT_1_ cut‐offs—for example, 18 in Taiwanese cohorts [15] versus 14 in an Indian study [13]—with performance further influenced by age, education, and cultural‐normative context [11, 16].

To explore whether demographic adjustment might partially mitigate this issue, we performed a post hoc exploratory calibration of S‐ANT_1_ in Taiwanese Hokkien speakers by adding three points for age > 70 years and/or education < 6 years. This exploratory Taiwanese‐calibrated S‐ANT_1_ showed a numerically higher AUROC than the original S‐ANT_1_, although the AUROC difference was not statistically significant by DeLong's test. We acknowledge that this score was derived from the demographic distribution of the same Taiwanese Hokkien‐speaking subgroup and is therefore vulnerable to optimism bias and overfitting. Although bootstrap internal validation was performed within this subgroup, the wide confidence interval and limited number of MHE events preclude firm conclusions regarding generalizability. Therefore, this calibrated score should be regarded as hypothesis‐generating and requires external validation in a larger independent Taiwanese Hokkien‐speaking cohort before clinical implementation. The sociolinguistic interpretation of the observed difference between Mandarin– and Taiwanese Hokkien‐speaking patients should be regarded as speculative. Primary spoken language may reflect not only language use but also age, education, literacy, and lifetime sociolinguistic exposure. However, bilingual proficiency, code‐switching, literacy, vocabulary knowledge, and lifetime language exposure were not directly measured in the present study. Therefore, these factors should be interpreted only as plausible explanations, not as confirmed mechanisms.

Our study also showed that serum IL‐6 levels were significantly elevated in MHE patients, supporting its potential role as an inflammatory biomarker in MHE assessment [8, 9]. However, the incremental value of serum IL‐6 appeared to differ by language subgroup and psychometric scoring approach. In Mandarin‐speaking patients, integrating ANT_1_ with serum IL‐6 showed a numerically higher AUROC, although this improvement was not statistically significant in pairwise DeLong comparisons. In Taiwanese Hokkien‐speaking patients, adding IL‐6 to the exploratory Taiwanese‐calibrated S‐ANT_1_ did not numerically improve AUROC. However, this absence of observed improvement should not be interpreted as evidence that IL‐6 has no incremental value. Given the small sample size, these subgroup findings should be interpreted cautiously, and larger studies are required to determine whether biomarker integration provides clinically meaningful improvement across different language groups. The IL‐6 cutoff identified in our cohort was lower than those reported in previous European studies. In particular, applying higher thresholds, such as 8 or 11 pg/mL, to our cohort would reduce sensitivity and miss a substantial proportion of MHE cases. This discrepancy may reflect differences in disease severity, cirrhosis etiology, outpatient versus referral cohort composition, and other cohort‐specific factors. Therefore, our IL‐6 cutoff should be regarded as exploratory and cohort‐specific, and serum IL‐6 thresholds require external validation before clinical implementation.

Our study has several limitations. First, the sample size was modest, particularly in the language‐stratified subgroups, and the findings require validation in larger multicenter cohorts. Second, primary spoken language was classified pragmatically as Mandarin or Taiwanese Hokkien based on self‐reported dominant daily language use and the language selected for ANT_1_ administration. However, this binary classification does not capture the continuous nature of bilingual proficiency, code‐switching, literacy, or lifetime language exposure in Taiwan. Future studies should incorporate validated bilingual‐dominance instruments, such as the Bilingual Language Profile [26], to more precisely quantify language exposure and language dominance. Third, patients with MHE were older than non‐MHE patients, and age may partly contribute to differences in psychometric performance. Although we performed age‐adjusted scoring, multivariable analysis, age‐stratified analysis, and exploratory demographic calibration, residual confounding cannot be excluded. Fourth, the exploratory Taiwanese‐calibrated S‐ANT_1_ was derived from the same Taiwanese Hokkien‐speaking subgroup and may therefore be vulnerable to optimism bias and overfitting. Although bootstrap internal validation was performed, the wide confidence interval and limited number of MHE events preclude firm conclusions regarding generalizability. Fifth, because PHES was included in the composite reference standard and ANT_1_/S‐ANT_1_ are also psychometric tests, the AUROC estimates for ANT_1_‐based measures may be affected by partial incorporation bias. To address this concern, we performed an exploratory sensitivity analysis using EEG‐defined MHE as the reference condition, which showed that ANT_1_, S‐ANT_1_, serum IL‐6, and the composite model retained moderate discriminatory performance. Nevertheless, because only 13 patients were EEG‐positive, this sensitivity analysis should be interpreted cautiously and requires validation in larger independent cohorts using neurophysiological or outcome‐based endpoints. Finally, our cohort showed lower median serum IL‐6 levels than the European cohorts of Montoliu [9] and Gairing [10]. Therefore, the IL‐6 cutoff identified in this study should be regarded as exploratory and cohort‐specific, and external validation is required before clinical implementation.

In conclusion, this proof‐of‐concept study suggests that primary spoken language may be associated with ANT_1_ performance in the assessment of MHE. In Mandarin‐speaking patients, integrating ANT_1_ with serum IL‐6 showed a numerically higher AUROC but did not demonstrate statistically significant superiority over either marker alone. In Taiwanese Hokkien‐speaking patients, an exploratory demographic calibration of S‐ANT_1_ showed potential improvement in performance. These findings should be interpreted as exploratory and hypothesis‐generating and require external validation before clinical implementation.

## Funding

This work was supported by CMRPG3P0761 and CMRPG3M1932 from Chang Gung Memorial Hospital (CMRP).

## Conflicts of Interest

The authors declare no conflicts of interest.

## Supporting information


**Table S1A:** Multivariable analysis on the association with MHE in total population.
**Table S1B:** Multivariable analysis on the association with MHE in Mandarin population.
**Table S1C:** Multivariable analysis on the association with MHE in Taiwanese‐Hokkien population.
**Table S2:** Age‐stratified diagnostic performance of ANT1, serum IL‐6, and the integrated ANT1 + IL‐6 model for MHE.
**Table S3:** Baseline characteristics according to primary spoken language.
**Table S4:** Diagnostic performance of cohort‐specific and previously reported serum IL‐6 cutoffs for MHE.
**Table S5:** Sensitivity analysis using EEG‐defined MHE as the reference condition.
**Table S6:** Bootstrap internal validation of the ANT_1−_based measures for detecting MHE in Taiwanese Hokkien‐speaking patients.

## Data Availability

The data that support the findings of this study are available from the corresponding author upon reasonable request.
