# Supplementary material for: A Proof‐of‐Concept Study of Language‐Stratified Assessment for Minimal Hepatic Encephalopathy: Integrating the Animal Naming Test and Serum IL‐6
Source: Kaohsiung J Med Sci. 2026 Aug 20:e70271. Online ahead of print. doi: 10.1002/kjm2.70271 (PMC13493613; doi:10.1002/kjm2.70271)
Supplement: Supplementary file 1 [file KJM2-9999-e70271-s001.docx]

**Supplementary Table 1A**. Multivariable analysis on the association with MHE in total population

| Variables | Univariable Analysis | | Multivariable Analysis | |
| --- | --- | --- | --- | --- |
|  | OR (95%C.I.) | P-Value | OR (95%C.I.) | P-value |
| Age | 1.076 (1.005-1.153) | 0.036 | 1.047 (0.968-1.132) | 0.248 |
| Education years | 0.922 (0.802-1.062) | 0.261 | 1.121(0.930-1.351) | 0.231 |
| Language |  |  |  |  |
| Mandarin | 0.514 (0.160-1.239) | 0.264 | 0.278 (0.059-1.314) | 0.106 |
| Taiwanese-Hokkien | Ref. |  |  |  |
| AN$T_{1}$score | 0.862 (0.761-0.978) | 0.021 | 0.796(0.654-0.969) | 0.023 |

**Supplementary Table 1B**. Multivariable analysis on the association with MHE in Mandarin population

| Variables | Univariable Analysis | | Multivariable Analysis | |
| --- | --- | --- | --- | --- |
|  | OR (95%C.I.) | P-Value | OR (95%C.I.) | P-value |
| Age | 1.067 (0.990-1.167) | 0.093 | 1.047(0.953-1.150) | 0.341 |
| Education years | 0.938 (0.784-1.128) | 0.482 | 1.229(0.932-1.620) | 0.144 |
| AN$T_{1}$score | 0.832 (0.674-0.969) | 0.016 | 0.744 (0.568-0.974) | 0.032 |

**Supplementary Table 1C**. Multivariable analysis on the association with MHE in Taiwanese-Hokkien population

| Variables | Univariable Analysis | | Multivariable Analysis | |
| --- | --- | --- | --- | --- |
|  | OR (95%C.I.) | P-Value | OR (95%C.I.) | P-value |
| Age | 1.056 (0.946-1.199) | 0.336 | 1.047(0.913-1.201) | 0.509 |
| Education years | 0.935 (0.747-1.131) | 0.470 | 1.021(0.783-1.330) | 0.879 |
| AN$T_{1}$score | 0.883 (0.682-1.074) | 0.226 | 0.868(0.648-1.164) | 0.345 |

**Supplementary Table 2**. Age-stratified diagnostic performance of ANT1, serum IL-6, and the integrated ANT1 + IL-6 model for MHE

| Age group | n | MHE, n (%) | AN$T_{1}$AUROC | IL-6 AUROC | Integrated AN$T_{1}+$IL-6 AUROC |
| --- | --- | --- | --- | --- | --- |
| <65 years | 40 | 7(17.50%) | 0.820 | 0.719 | 0.827 |
| ≧65 years | 25 | 9(36.00%) | 0.528 | 0.951 | 0.813 |

**Supplementary table 3**. Baseline characteristics according to primary spoken language

| Variables | Mandarin speakers | Taiwanese-Hokkien speakers | P-value |
| --- | --- | --- | --- |
| Number | 44 | 21 |  |
| MHE, n (%) | 9 (20.45%) | 7 (33.33%) | 0.260 |
| Male sex, n (%) | 29 (65.91%) | 14 (66.67%) | 0.952 |
| EEG slowing, n (%) | 8 (18.18%) | 5 (23.81%) | 0.742 |
| Etiology of cirrhosis |  |  | 0.265 |
| HBV | 27 (61.36%) | 10 (47.62%) |  |
| HCV | 7 (15.91%) | 2 (9.52%) |  |
| Alcohol | 8 (18.18%) | 9 (42.86%) |  |
| NASH | 1 (2.27%) | 0 (0.00%) |  |
| Others | 1 (2.27%) | 0 (0.00%) |  |
| Age, years | 60.00 (52.00-66.00) | 65.00 (60.00-70.00) | 0.054 |
| Education years | 12.00 (9.00-12.00) | 9.00 (6.00-12.00) | 0.197 |
| MELD score | 9.00 (8.00-15.00) | 12.00 (10.00-14.00) | 0.299 |
| PHES | 0.00 (-2.00-1.00) | -1.00 (-4.00-0.25) | 0.135 |
| AN$T_{1}$score | 15.00 (12.00-21.25) | 19.00 (16.00-21.00) | 0.121 |
| S-AN$T_{1}$score | 16.00 (14.00-21.25) | 19.00 (17.00-22.00) | 0.064 |
| Exploratory Taiwanese- calibrated S-ANT_1_ | 16.00 (13.25-22.00) | 19.00 (18.00-22.00) | 0.052 |
| MMSE | 29.00 (26.00-29.00) | 27.00 (25.00-29.00) | 0.076 |
| IL-6, pg/mL | 3.21 (2.00-5.70) | 4.20 (3.12-6.78) | 0.158 |

Continuous variables were presented as median (interquartile range) and compared using the Mann–Whitney U test when the Shapiro–Wilk test indicated non-normal distribution. Categorical variables were compared using the chi-square test or Fisher’s exact test, as appropriate. Fisher’s exact test was used when the minimum expected cell count was <5.

**Supplementary Table 4**. Diagnostic performance of cohort-specific and previously reported serum IL-6 cutoffs for MHE

| Serum IL-6 cutoff | Sensitivity | Specificity | PPV | NPV |
| --- | --- | --- | --- | --- |
| IL-6 ≧4.1 pg/mL | 0.875 | 0.694 | 0.464 | 0.919 |
| IL-6 ≧7 pg/mL | 0.500 | 0.898 | 0.615 | 0.846 |
| IL-6 ≧8 pg/mL | 0.438 | 0.898 | 0.583 | 0.830 |
| IL-6 ≧11 pg/mL | 0.125 | 0.939 | 0.400 | 0.767 |

PPV, positive predictive value; NPV, negative predictive value. The 4.1 pg/mL threshold represents the cohort-specific Youden cutoff. Fixed thresholds of 7, 8, and 11 pg/mL were evaluated based on previously reported or suggested IL-6 cutoffs. MHE was defined using the composite reference standard of abnormal PHES and/or visually assessed EEG slowing.

**Supplementary Table 5**. Sensitivity analysis using EEG-defined MHE as the reference condition

| **Predictor** | **AUROC** | **95% CI** | **P-value** |
| --- | --- | --- | --- |
| **ANT_1_** | 0.737 | 0.594-0.879 | 0.009 |
| **S-ANT_1_** | 0.706 | 0.547-0.865 | 0.022 |
| **Exploratory Taiwanese- calibrated S-ANT_1_** | 0.761 | 0.611-0.911 | 0.004 |
| **MMSE** | 0.699 | 0.526-0.872 | 0.027 |
| **Serum IL-6** | 0.723 | 0.594-0.853 | 0.013 |
| **Composite model** | 0.766 | 0.630-0.903 | 0.003 |

AUROC, area under the receiver operating characteristic curve; CI, confidence interval;

EEG-defined MHE was defined as visually assessed EEG slowing compatible with HE, regardless of PHES status. Because only 13 patients were EEG-positive, this sensitivity analysis was performed in the total cohort only and was not further stratified by primary spoken language.

**Supplementary Table 6**. Bootstrap internal validation of the ANT_1-_based measures for detecting MHE in Taiwanese Hokkien-speaking patients

| Predictors | Apparent AUROC | Bootstrap-corrected-AUROC | | | 95%CI |
| --- | --- | --- | --- | --- | --- |
| AN$T_{1}$ | 0.679 | | 0.668 | 0.467 – 0.873 | |
| Original S-AN$T_{1}$ | 0.617 | | 0.590 | 0.353 – 0.788 | |
| Exploratory Taiwanese-calibrated S-AN$T_{1}$ | 0.776 | | 0.773 | 0.578 – 1.000 | |

AUROC, area under the receiver operating characteristic curve; CI, confidence interval; MHE, minimal hepatic encephalopathy.
